# Hyponatremia is a potential predictor of progression in radiation-induced brain necrosis: a retrospective study

**DOI:** 10.1186/s12883-018-1135-z

**Published:** 2018-08-29

**Authors:** Huan Liao, Zhuoting Zhu, Xiaoming Rong, Hongxuan Wang, Ying Peng

**Affiliations:** 1 Department of Neurology, Sun Yat-sen Memorial Hospital, Sun Yat-Sen University, No. 107 West Yanjiang Road, Guangzhou, 510120 China; 2 State Key Laboratory of Ophthalmology, Zhongshan Ophthalmic Center, Sun Yat-sen University, Guangzhou, China; 3 Guangdong Provincial Key Laboratory of Malignant Tumor Epigenetics and Gene Regulation, Sun Yat-sen Memorial Hospital, Sun Yat-sen University, Guangzhou, China

**Keywords:** Hyponatremia, Radiation-induced brain necrosis

## Abstract

**Background:**

To investigate the prognostic value of hyponatremia, defined as serum sodium level < 135 mEq/L, in radiation-induced brain necrosis (RN) patients.

**Methods:**

We performed a retrospective analysis of the RN patients (The patients included in our study had a history of primary cancers including nasopharyngeal carcinoma/glioma/oral cancer and received radiotherapy previously and then were diagnosed with RN) treated in Sun yat-sen Memorial Hospital from January 2013 to August 2015. Patients without cranial magnetic resonance imaging (MRI) scan and serum sodium data were excluded. Progression was identified when the increase of edema area ≥ 25% on the MRI taken in six months comparing with those taken at the baseline. Factors that might associate with prognosis of RN were collected. Multivariable logistic regression analyses were used to identify potential predictors.

**Results:**

We total included 135 patients, 32 (23.7%) of them with hyponatremia and 36 (26.7%) with RN progression. Percentage of progression was roughly three fold in hyponatremia patients compared with nonhyponatremia patients (53.1% versus 18.4%), translating into a 5-fold increased odds ratio (*P* <  0.001). Multivariable analyses identified hyponatremia as a potential predictor of progression (OR, 4.82; 95% CI [1.94–11.94]; *P* = 0.001).

**Conclusions:**

Hyponatremia was identified as a potential predictor for the progression of patients with RN. Hyponatremia management in patients with RN should be paid much more concern in clinical practice.

## Background

Radiotherapy is the standard treatment for nasopharyngeal carcinoma (NPC) and various brain tumors. NPC is the third most common malignant tumor in men in certain regions of East Asia, with an incidence of 15/100,000 to 50/100000 [1]. Moreover, since radiotherapy is invariably associated with radiation exposure of surrounding healthy tissues [2], nearly 100,000 primary and metastatic brain neoplasm patients/year survive long enough (> half a year) to suffer radiation-induced brain necrosis (RN) in the US [3]. RN is a progressive disease, rendering impairments in attention, memory and executive function, which results in decreased patient quality of life [3].

There is consistent evidence demonstrating that hyponatremia not only acts as risk factors in various diseases such as cancer [4] and heart failure [5], but also has close relationship with central nervous system (CNS) diseases, such as stroke [6], subarachnoid hemorrhage [7, 8] and meningitis [9, 10], through inducing longer hospital stay, increased mortality, and raised complications [11–14]. In neurointensive care, it is usually the development of delayed cerebral infarctions, seizures, and cerebral edema that connects hyponatremia to prognosis [6]. Documentaries also suggest that hyponatremia probably serves as an onlooker reflecting the severity of diseases since it might be preexisting [6, 15, 16], which might be induced by cerebral salt wasting syndrome(CWS) and the syndrome of inappropriate antidiuretic hormone secretion(SIADH) [13, 17, 18].

Whereas, for RN, whether hyponatremia is related to the prognosis of RN has not been addressed. In this study, by facilitating a comprehensive coverage of patient characteristics, we aimed to clarify factors associated with prognosis of RN.

## Methods

### Patient selection

The study was approved by the Institutional Review Board of Sun yat-sen Memorial Hospital. We retrospectively collected the data of patients diagnosed with RN (The patients included in our study had a history of primary cancers including nasopharyngeal carcinoma/glioma/oral cancer and received radiotherapy previously and then were diagnosed with RN) and treated at Department of Neurology, Sun Yat-Sen Memorial Hospital from January, 2013 to August, 2015. The eligible criteria were listed as following: (1) Patients diagnosed with RN between January, 2013 and August, 2015 in the Department of Neurology, Sun Yat-Sen Memorial Hospital; (2) Patients with cranial MRI scan at baseline (the date of the first MRI performed for the diagnosis of RN for the patients in Sun Yat-Sen Memorial Hospital between January, 2013 and August, 2015) and six-month follow-up; (3) Available data of serum sodium during the period between the time of baseline and six-month follow-up.

### Clinical details

Patients’ characteristics including age, sex, in-hospital days, history of hypertension, epilepsy, dyslipidemia, cerebral infarction, pulmonary infection, paralysis of cranial nerves, nasopharyngeal carcinoma, glioma, oral cancer and baseline result of cranial MRI were retrieved from our institutional prospective electronic medical records. Serum sodium values were measured by use of Ion Selective Electrodes with automated specimen dilution (Cobas Integra 800, Roche) and the lowest values within the time period mentioned above were selected for analysis. Hyponatremia was defined as serum sodium values less than 135 mmol/L according to Hyponatremia Guidelines in Europe in 2014 [19] and was corrected by using hypertonic saline infusions (NaCl, 3%) or 0.9% sodium infusions aiming at an increase of ≤8 mg/dL per 24 h. In addition, all patients were treated with a conventional corticosteroid regimen as described in our prior study [20].

### MRI acquisition and analysis

Diagnosis of RN was made by clinical history and MRI performance (Sonata; 1.5 T; Siemens), which included T1-weighted gadolinium contrast-enhanced and T2-weighted image. The MRI recognition of RN was referred to a prior study [21]. Typically, the most common feature of MRI in patients with RN is that the appearance of focal necrosis and finger like edema displays low signal intensity on T1WI while high signal on T2WI. We draw the edge of the maximum area of each edema manually which was then calculated automatically by software Volume Viewer 2(GE, AW Suite 2.0, 6.5.1.z). Two neuroradiologists who were blinded to clinical data reviewed the scans independently. Particularly, edema area was counted as the mean value of two measurements. Moreover, in cases of discrepancies, a second consensus analysis was made by the third author. Follow-up MRIs at six months were used to evaluate the end-point. The end-point is the edema progression occurrence, based on our prior methods [20]. Briefly, the edema progression occurrence was identified when the increase of edema area > 25%.

### Statistical analysis

Student’s t test (presented as mean ± SD) or Mann-Whitney test (median, range) was used to compare continuous variables, while Pearson Chi-square or Fisher’s exact test was used for the comparison of categorical data where appropriate. Multivariable logistic analyses were calculated to investigate independent risk factors for progression. Parameters reaching a statistical trend in univariable analysis (*P* <  0.30) were included into the multivariable models. Collinearity was examined using variance inflation factors (VIF) procedure. Statistical significance was defined as a *P* value < 0.05 at 2-sided. All statistical analyses were performed with SPSS version 22.0 (SPSS Inc).

## Results

**Fig. 1:**
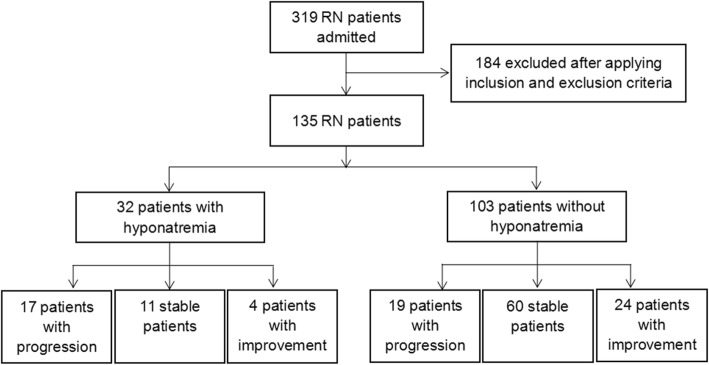
Study flow chart

A total of 135 patients were included in our study (Fig. 1), with a mean age of 50.9 ± 10.0 years old. 103 patients (76.3%) were male and 32 (23.7%) were female. Among the patients, 126 (93.3%) had a history of nasopharyngeal carcinoma, 8 (6.0%) had glioma and 1 (0.7%) had oral cancer. The median timing of the lowest concentration was one month after the first MRI performed. The mean of the lowest serum sodium values within the six-month period of each patients were 136.7 ± 5.1 mmol/L. Out of 135 patients enrolled for our analysis, 32 patients (23.7%) had hyponatremia and 36 (26.7%) occurred edema progression (increase of edema area > 25%) within a six-month follow-up.

Table 1 shows the comparison of clinical characteristics between patients with and without hyponatremia. Hyponatremia patients were showing statistical trends to have more male gender (87.5% versus 72.8%, *P* = 0.088) and more frequent existing of pulmonary infection (12.5% versus 3.0%, *P* = 0.093). In other aspects of clinical characteristics, there were no significant differences between these two groups.

**Table 1:** Comparison of characteristics between patients with or without hyponatremia

Characteristics	Hyponatremia *n* = 32	Nonhyponatremia *n* = 103	*P*-Value
Serum sodium, mmol/L (mean ± SD)	129.3 ± 4.3	139.0 ± 2.4	< 0.001
Age, yrs. (mean ± SD)	52.0 ± 9.8	50.5 ± 10.0	0.465
Male	28 (87.5%)	75 (72.8%)	0.088
In-hospital days (M)	8.3 (4–20)	7.5 (1.8–23)	0.546
Hypertension	7 (21.9%)	16 (15.5%)	0.405
Epilepsy	3 (9.4%)	15 (14.6%)	0.648
Dyslipidemia	4 (12.5%)	11 (9.7%)	1.000
Cerebral infarction	5 (15.6%)	8 (7.8%)	0.330
Pulmonary infection	4 (12.5%)	3 (3.0%)	0.093
Paralysis of cranial nerves	7 (21.9%)	16 (15.5%)	0.405
Nasopharyngeal carcinoma	31 (96.9%)	95 (92.2%)	0.607
Glioma	1 (3.1%)	7 (6.8%)	0.734
Oral cancer	0 (0%)	1 (1.0%)	1.000
Baseline T1-weight gadolinium-contrast area (cm2)(mean ± SD)	5.1 ± 2.3	5.3 ± 3.8	0.810
BaselineT2-weighted area (cm2) (mean ± SD)	10.6 ± 5.5	10.4 ± 6.6	0.843

*M* median, *SD* standard deviation

Table 2 displays the clinical characteristics of the patients divided into 2 groups according to the presence of edema progression. Patients whose cranial MRI indicating progression were more likely to have lower serum sodium concentration (132.9 ± 6.2 mmol/L versus 138.1 ± 3.8 mmol/L, *P* <  0.001) and higher incidence of medical history on hypertension (27.8% versus 13.1%, *P* = 0.045).

**Table 2:** Comparison of characteristics between patients with or without progression

Characteristics	With progression *n* = 36	Without progression *n* = 99	*P*-Value
Serum sodium, mmol/L (mean ± SD)	132.9 ± 6.2	138.1 ± 3.8	< 0.001
Age, yrs. (mean ± SD)	52.7 ± 11.0	50.2 ± 9.5	0.207
Male	27 (75%)	76 (76.8%)	0.831
In-hospital days (M)	8.3 (4.5–23)	7.3 (1.8–22)	0.119
Hypertension	10 (27.8%)	13 (13.1%)	0.045
Epilepsy	4 (11.1%)	14 (14.1%)	0.864
Dyslipidemia	3 (8.3%)	12 (12.1%)	0.757
Cerebral infarction	6 (16.7%)	7 (7.1%)	0.180
Pulmonary infection	4 (11.1%)	3 (3.0%)	0.152
Paralysis of cranial nerves	7 (19.4%)	16 (16.2%)	0.654
Nasopharyngeal carcinoma	33 (91.7%)	93 (94.0%)	0.938
Glioma	3 (8.3%)	5 (5.0%)	0.762
Oral cancer	0 (0%)	1 (1.0%)	1.000
Baseline T1-weight gadolinium-contrast area (cm2)(mean ± SD)	4.5 ± 2.4	5.5 ± 3.8	0.153
BaselineT2-weighted area (cm2) (mean ± SD)	9.3 ± 5.9	10.8 ± 6.4	0.200

*M* median, *SD* standard deviation

Percentage of progression was roughly three fold in hyponatremia compared with nonhyponatremia patients (53.1%; *n* = 17 versus 18.4%; *n* = 19), translating into a 5-fold increased odds ratio (95% CI, [2.13, 11.77]; *P* <  0.001). Multivariable analyses identified hyponatremia as an independent risk factor of progression (OR, 4.82; 95% CI, [1.94, 11.94]; *P* = 0.001), after adjustment with covariables including age, in-hospital days, history of hypertension, cerebral infarction, pulmonary infection and edema area in baseline MRI scan, which were found no collinearity existing (Table 3).

**Table 3:** The logistic regression for the relationship between progression of RN and factors

	Progression	Unadjusted		Adjusted	
		OR (95% CI)	*P*-Value	OR (95% CI)	*P*-Value
Hyponatremia (*n* = 32)	17 (53.1%)	5.01(2.13; 11.77)	< 0.001	4.82(1.94; 11.94)	0.001
Age	–	–		1.01(0.97; 1.06)	0.657
In-hospital days	–	–		1.05(0.95; 1.16)	0.350
Hypertension (*n* = 23)	10 (43.5%)	–		2.17(0.71; 6.60)	0.172
Cerebral infarction (*n* = 13)	6 (46.2%)	–		2.04(0.47; 8.82)	0.342
Pulmonary infection (*n* = 7)	4 (57.1%)	–		3.46(0.53; 22.43)	0.194
T1-weight gadolinium-contrast area	–	–		1.10(0.70; 1.73)	0.672
T2-weighted area	–	–		0.88(0.69; 1.12)	0.299

*OR* odds ratio, *CI* confidence interval

## Discussion

To the best of our knowledge, this is the first retrospective study to document the association between hyponatremia and outcome of cranial MRI in RN patients. Our findings show that hyponatremia is a potential predictor of progression of RN.

It’s very common to have hyponatremia among inpatients and outpatients, with prevalence ranging from 11 to 28.2% [22]. In our study, we found 23.7% of the enrolled RN patients with hyponatremia. Hyponatremia has acquired broad recognition as a predictor being associated with poor outcome in various diseases [23, 24]. Two main hypothetical mechanisms of hyponatremia impacting on prognosis in disorders of CNS were SIADH and CSW [25]. Excessive antidiuretic hormone (ADH) release causing renal water reabsorption and expansion of the extracellular fluid volume (ECF) was the primary pathogenic mechanism underlying SIADH. When hypothalamus and pituitarium were under pressure or got injured, ADH would be secreted abnormally, causing hyponatremia. Meantime, the mechanism by which RN leads to renal salt wasting is also poorly understood. The most probable process involved central elaboration of a circulating natriuretic factor and/or disruption of neural input into the kidney. Atrial natriuretic peptide (ANP), brain natriuretic peptide (BNP), c-type natriuretic peptide (CNP) and ouabainlike compound (OLC) had been demonstrated to play a vital role in CSW [26–30], whose releasing also was related to the disruption of hypothalamus and pituitarium. Of note, it is important to make an accurate diagnosis because the treatment of each condition is quite different. Fluid restriction is the treatment of choice in patients with SIADH while vigorous salt replacement is required in patients with CSW. Existing data suggested that fluid restriction was very likely to worsen the underlying neurological condition in the setting of CSW, even cause cerebral infarction [31], acute symptomatic seizures [32] or death. Moreover, the clinical symptoms of vomiting and headache, were often regarded as the results of intracranial hypertension caused by brain edema in RN patients, thus mannitol was usually applied to alleviate the symptom. Nevertheless, in fact, the symptoms could not get remission sometimes, which implied that the symptoms were not caused by brain edema, but very likely by hyponatremia [33]. The information above concludes that, on one hand, when doing irradiation therapy for the primary tumors, avoiding radiation exposure of hypothalamus and pituitarium if possible may effectively reduce the occurrence of hyponatremia from the source [34, 35]. On the other hand, more proper dehydrants instead of those dehydrants which can cause hyponatremia should be chosen when dealing with brain edema of RN. Better management of hyponatremia in RN patients needs more attention.

There were several studies indicating that inflammatory cytokines such as Interleukin 6 (IL-6) [36] and Interleukin 1 beta (IL-1β) [37] play vital roles in the nonosmotic release of ADH. Under inflammatory conditions, this mechanism may be responsible for the occurrence of hyponatremia [38]. Interestingly, the mechanism of RN involves damage to the immune system [39]. In addition, previous studies found that after radiation, microglia in mouse brain tissue could secrete several inflammatory cytokines including IL-6, IL-1β, tumor necrosis factor alpha (TNF-α) and cyclooxygenase-2 (COX-2) [40]. Therefore, we speculate disorder of immune system could render the development of hyponatremia in patients with RN, which should be demonstrated by further studies.

However, despite these postulated pathophysiological considerations of hyponatremia-induced impacts on prognosis, it remains unsolved if hyponatremia merely stands for an onlooker of diseases, that is, preexisting polymedication or comorbidity mirroring worse overall status, which itself maybe responsible for poor prognosis. For instance, hyponatremia was independently associated with anemia on hospital admission, mentioning that hyponatremia was probably preexisting condition rather than developing acutely [6]. Accordingly, in our study, hypertension was also found to be associated with progression of RN. This need to be paid more attention because all hyponatremia patients present with hyponatremia on admission rather than developed it during the course of clinical management [6]. In addition, no existing evidence across a variety of disciplines and diseases demonstrated that correcting hyponatremia could result in better clinical end points so far [41].

Our study firstly demonstrated the association between hyponatremia and prognosis of RN, broadening the area of factors impacting on RN outcome and thus offered a new target on improving the prognosis in clinical practice. Nevertheless, this study still had some limitations. First, retrospective design left the unanswered question why hyponatremia patients presented increased progression of RN. Second, we did not collect the unavailable information regarding the irradiation strategy (radiation fields together with radiation dosages and schedule), though irradiation strategy has been demonstrated to be associated with occurrence rate of RN [42], which may also have impacts on the outcome of the patients. Third, we could not exclude other causes besides radiation necrosis that might also lead to hyponatremia in RN patients. For example, Table 1 shows a 2× higher frequency of cerebral infarction and a 4× higher frequency of pulmonary infarction associated with hyponatremia. Probably, the limited number of patients studied explains that these observations are not significant. In addition, results may have been biased by excluded patients with uncompleted data. Moreover, the data in the paper failed to answer that when and how often to use hyponatremia detection, and once hyponatremia is detected when the next MRI should be done. Additionally, our data showed that the median timing of the lowest serum sodium concentration was one month after the first MRI performed. The potential explanation maybe that one month after the first MRI performed, patients suffered from bad symptoms (which might be a sign of RN progression) and had to go to hospital to have medical check including blood examination which showed low concentration of sodium including hyponatremia, furtherly surpporting that hyponatremia is a potential predictor for the progression of patients with RN. However, more studies are warranted to confirm and explain this. Lastly, MR spectroscopy is a potential better choice to distinguish recurrent tumor and radiation injury in patients previously radiated for brain neoplasm [43]. However, since the MR spectroscopy data of the enrolled patients wasn’t available, we had to choose conventional MR imaging.

## Conclusions

In summary, hyponatremia is a common phenomenon in the hospitalized RN patients and acts as a potential predictor of progression, which has clinical significance that doctors need to pay more attention to the management of hyponatremia in RN patients. Large and prospective studies are needed to verify these findings and provide further evidence.

## Abbreviations

ADH: Antidiuretic hormone
CI: Confidence interval
CWS: Cerebral salt wasting syndrome
M: Median
MRI: Magnetic resonance imaging
OR: Odds ratio
RN: Radiation-induced brain necrosis
SD: Standard deviation
SIADH: The syndrome of inappropriate antidiuretic hormone secretion
VIF: Variance inflation factors
